# Rotating night work, lifestyle factors, obesity and promoter methylation in *BRCA1* and *BRCA2* genes among nurses and midwives

**DOI:** 10.1371/journal.pone.0178792

**Published:** 2017-06-08

**Authors:** Beata Peplonska, Agnieszka Bukowska, Edyta Wieczorek, Monika Przybek, Shanbeh Zienolddiny, Edyta Reszka

**Affiliations:** 1 Department of Environmental Epidemiology, Nofer Institute of Occupational Medicine, Lodz, Poland; 2 Department of Molecular Genetics and Epigenetics, Nofer Institute of Occupational Medicine, Lodz, Poland; 3 National Institute of Occupational Health, Oslo, Norway; Ohio State University Wexner Medical Center, UNITED STATES

## Abstract

Some recent evidence suggests that environmental and lifestyle factors may modify DNA methylation. We hypothesized that rotating night work and several modifiable factors may be associated with the methylation of the promoter regions within two tumor suppressor and DNA repair genes: *BRCA1* and *BRCA2*. The methylation status of *BRCA1* and *BRCA2* was determined via qMSP reactions using DNA samples derived from blood leucocytes of 347 nurses and midwives working rotating nights and 363 working during the days. The subjects were classified into unmethylated vs methylated *BRCA*1 and *BRCA*2 when the methylation index was 0% or >0%, respectively. The adjusted odds ratios with 95% confidence intervals were calculated for night work status, smoking, obesity, physical activity and alcohol drinking. Current night shift work or night work history was not associated with methylation status of the promoter sites within *BRCA1* and *BRCA2* genes. We observed weak associations between smoking and the methylation status of *BRCA*1 with OR = 1.50 (95%CI: 0.98–2.29) for current smoking, OR = 1.83, 95CI: 1.08–3.13 for smoking longer than 31 years, and 0.1>p>0.05 for trends for the number of cigarettes per day, smoking duration and packyears. In conclusion, no links between night shift work and methylation of the promoter region within the *BRCA*1, and *BRCA2* genes were observed in this exploratory analysis. The findings of our study weakly support the hypothesis that smoking may contribute to epigenetic events.

## Introduction

Breast cancer is the most prevalent cancer among women worldwide. The etiology of breast cancer has not been fully explained; however, both the genetic and environmental factors are postulated to be playing a role. Of the genetic factors, the inherited germline mutations in two genes: *BRCA1* and *BRCA2*, are the well-established risk factors of breast cancer, explaining approximately two percent of breast cancer cases [1] (up to 7 percent among Polish women diagnosed before age 50[2]). These two tumor suppressor genes are involved in genome stability, DNA repair and cell cycle control [3].

Of the modifiable risk factors, obesity after menopause [4] and alcohol drinking [5] have been recognized as potentially contributing to breast cancer, while physical activity has been proven to play some part in reducing breast cancer risk [6]. Epidemiological evidence has also implied a potentially causative role of active smoking, particularly long-term heavy smoking [7], and when initiated before first birth [8].

One of the occupational exposures that have been classified as probably carcinogenic is the shift work leading to circadian rhythm disruption [9]. A number of epidemiological studies were focused on a possible association between night shift work and breast cancer risk [10–13]. The meta-analysis based on 12 case-control studies reported a 9% increase in the risk of breast cancer per 5 years of night work [10]. However, no increased risk of breast cancer was observed in the some recent prospective cohort studies [14]. Thus the link between night shift work and breast cancer risk still remains disputable.

One of the proposed mechanisms underlying the increased risk of breast cancer among night shift workers is a decrease in melatonin synthesis [15–17] and an increase in reproductive hormones synthesis in response to light-at-night [18]. It has also been postulated that shift work may contribute to unhealthy changes in lifestyle [19], such as more common smoking [20–34] and alcohol consumption [21,23,24,26,30,33,35–38] as well as lower physical activity [27,33,35,39] and poor dietary habits [40], which may increase cancer risk. Moreover, night workers were found to be more likely to become obese than day workers [41,42].

Although the idea of shift work as being probably carcinogenic to humans is gaining widespread attention, there is a lack of experimental mechanistic evidence linking shift work to breast cancer.

Moreover, the biological and molecular mechanisms of shift work exposure have not been fully explored. Only recently have some *in vivo* animal models of human shift work been used to investigate the possible association between shift work and adverse health effects [43]. Epigenetic changes in the genome have been proposed as one of the biologically plausible mechanisms [44–46]. A major epigenetic mechanism that may affect gene expression in the disease is the changes in the methylation level of 5-methylcytosine (5meC) in the regulatory regions (i.e. promoter, enhancer) of the genes. This may lead to an altered transcription of the regulatory genes, which can result in malignant cellular transformation.

Several studies have already investigated genome-wide CpG island methylation of the protein-coding genes and the regulatory microRNA (miRNA) genes [44,47–49]*Wee EJ*, *Peters K*, *Nair SS*, *Hulf T*, *Stein S*, *Wagner S*, *Bailey P*, *Lee SY*, *Qu WJ*, *Brewster B*, *et al*. *Oncogene*. *2012 Sep 20; 31(38)*:*4182–95* according to the shift work status. Their findings suggest that a long-term night shift work exposure may lead to methylation-dependent downregulation of the protein-coding genes and miRNA genes, which in turn may lead to the downregulation of several biological pathways that are important in breast carcinogenesis, such as DNA damage response and repair, oxidative stress response, inflammation, and immunomediated antitumor activity. Changes in promoter methylation in the CLOCK gene were also reported among long-term shift workers [44], which was consistent with the findings on a low level of the *CLOCK* methylation in breast cancer patients [50]. In vitro assays displayed a network of transcripts with an altered expression following *CLOCK* or *CRY2* gene knockdown, including the genes involved in breast carcinogenesis [50,51].

The evidence for the role of environmental and lifestyle factors in DNA methylation has been mounting. Changes in the methylation pattern of the promoter within tumor suppressor genes have also been observed, with suggestive evidence supporting the hypothesis that not only the inherited mutations in the genes, but also their functional inactivation caused by the epigenetic events such as hypermethylation i.e. aberrant methylation of the CpG islands in the promoter region, may play a role in the etiology of breast cancer [52]. Some evidence points out that the epigenetic regulation of the *BRCA*1 gene may contribute to breast cancer risk [52,53].

Therefore, we hypothesized that the rotating night work and such modifiable factors as cigarette smoking, alcohol drinking, physical activity, and obesity, may be associated with the methylation of the promoter regions within two tumor suppressor and DNA repair genes: *BRCA1* and *BRCA2*. To examine this hypothesis, we investigated associations between the methylation status of the CpG island in the promoter of the *BRCA1* and *BRCA2* genes and the lifestyle factors, using data obtained from a cross-sectional study on nurses and midwives.

## Material and methods

This cross-sectional study was described elsewhere [34]. In short, the study included 725 nurses and midwives (84% of those eligible), aged 40–60 years, who were employed as a nurse or midwife in public health care settings in Lodz, Poland. The study participants were recruited based on the data derived from the Regional Registry of the Chamber of Nurses and Midwives in Lodz. The nurses currently working night shifts (n = 347) were employed according to the fast rotating system (12 h long shifts), with a night shift followed by a day off. Night duties started at 7 p.m. and ended at 7:00 a.m. while day shifts started at 7 a.m. and ended at 7 p.m., with no evening shifts. There were no permanent night workers in the study population.

Day workers (n = 363) worked mostly in the outpatient clinics and the shift usually lasted 7.5 hours, between 7 a.m. and 4 p.m. Among the subjects currently working day shifts, the majority had some history of night shift work, but most of them (83%) had resigned from night shifts more than five years before the onset of the present study.

A structured questionnaire was administered during in-person interviews that were carried out during the years 2008–2010, to elicit information on occupational history, demographics, medical and reproductive history, hormone use, physical activity, smoking, alcohol use, and diet; the latter based on the food frequency questionnaire (FFQ). Questions were asked about current cigarette smoking, and the amounts smoked within 5 years prior to the project; with the age categories starting at age <15 years. With regard to alcohol consumption, we asked questions about the number of glasses of beer (0.5 l), wine (100ml) or vodka (50ml) drunk on average per day (week, month or year) over the period of the preceding year, and we also asked about the number of alcoholic beverages drunk on average, by the age categories starting from the age of 15–17 years. Anthropometric measurements of body weight, height, waist and hip circumference were performed by trained nurses, and body mass index (BMI in kg/m^2^), and waist to hip ratio (WHR) were calculated.

Physical activity was assessed according to the International Physical Activity Questionnaire (IPAQ) [54], that included questions about four physical activity domains: leisure-time, occupational, transport-related and household activities. The details were described previously [55]. Based on the IPAQ guidelines, we calculated the MET-hours per week (MET—metabolic equivalent ratio of the metabolic rate to a standard resting metabolic rate of 1) for each category of physical activity. The total physical activity was calculated by summing the scores for all the four domains. Blood samples were collected from 710 women.

Between the years 2014 and 2015, the first follow-up was carried out among 632 women participants (87.2% of the original study population). At that time we collected information about the subjects’ diet, using the FFQ. Trained interviewers asked the participants about the frequency and amount of a usual intake of 151 nutritional items as well as about micronutrient and vitamin intake. To help the respondents assess the weight of the consumed foods, a photo book of food products and dishes was used, with pictures showing food items of different size and estimated weight attributed to each item in each size.

Both the original study and the follow-up were approved by the ethical institutional review board at the Nofer Institute of Occupational Medicine. A signed informed consent was obtained from each participant for each phase of the study.

### Laboratory methods

Blood samples were collected into S-Monovette® heparinized test tubes in the morning hours (06:00–10:00 a.m.). The samples were preserved immediately after being delivered to the lab and stored at -20°C until DNA isolation. Genomic DNA was isolated from 710 whole blood samples using QIAamp DNA Blood Mini Kit (Qiagen), according to the manufacturer’s instructions.

The promoter region with the transcriptional start site of *BRCA1* and *BRCA2* was analyzed using DBTSS database (http://dbtss.hgc.jp) for further CpG island identification. Chemical modification of 500 ng of genomic DNA isolated from whole blood was performed with the use of Cells-to-CpG™ Bisulfite Conversion Kit (Thermo Fisher Scientific, Waltham, MA, USA). DNA methylation was analyzed using quantitative methylation-specific real-time PCR assay (qMSP) with FastStart Essential DNA Probes Master (Roche, Basel, Switzerland). Primers and MethyLight dual-labelled probe were designed using Beacon Designer 7.01 (PREMIER Biosoft Int., Palo Alto, CA, USA). *BRCA1* and *BRCA2* amplicons covered 11 and 12 CpG dinucleotides, respectively. The qMSP*BRCA1* oligonucleotides were as follows: qMSP: 5’-GTATTTTGAGAGGTTGTTGTTTAGC-3’ (sense), 5’-CGTCCAAAAAATCTCAACGA-3’ (antisense) and 5’FAM-ACGCCGCGCAATCGCAATTTTAAT-3’BHQ1 (probe); qMSP*BRCA2–*5’-TTGAGAAATATTCGTAGCGGTTTATTTAGG-3’ (sense), 5’-CTAACCACGTAACGCCGTAACG-3’ (antisense) and 5’FAM-CACGCAACACACGCACCACCCGAAA-3’BHQ1 (probe). The qMSP was performed with internal reference genes *ACTB* and *MYOD* [56]. The Primers and the probe of the internal reference gene were located in the area without CpG nucleotides, thus amplifying the modified *ACTB* and *MYOD* gene independently of the methylation status of CpG nucleotides.

Water blanks were included in each plate to check possible contamination. Positive (fully CpG-methylated human genomic DNA), (NEB, Ipswich, MA, USA) and negative (5-Azadc treated human genomic DNA) (NEB, Ipswich, MA, USA) DNA controls were included in the methylation analysis. All the samples of 2μl of converted DNA were amplified with 0.6μM of primers and 0.2μM of probe in a 20μl reaction assay. To determine inter-assay variability, the qMSP for randomly selected samples was repeated. Inter-assay coefficients of variability (CV) were below 18% and intra-assay CV was below 8%. qMSP reactions were carried out on a 96-well plate in LC96 (Roche, Basel, Switzerland).

To calculate the methylation status of *BRCA1 and BRCA2*, reference gene-normalized relative methylation quantification, expressed as fully methylated reference (PMR—%), was used [56]. Briefly, the Ct values of the gene of interest were compared with the Ct values of the internal reference gene: PMR = 100% x 2^-ΔΔCt^ (ΔΔCt = (Ct of target gene in sample–Ct of control gene in sample)—(Ct of target gene in methylated control–Ct of control gene in methylated control).

### Statistical methods

Arithmetic means, with standard deviations and frequencies of the basic characteristics were calculated in the total population and according to the *BRCA1* and *BRCA2* methylation status.

To explore the association between lifestyle factors, current rotating night shift work and its duration and the *BRCA*1 or *BRCA*2 methylation status, a logistic regression model was used. The population was dichotomized into unmethylated vs methylated *BRCA*1 and *BRCA*2 when the methylation index was 0% or >0% respectively. *Apriori* list of the potential confounders was specified, and the subjects’ age and folate intake were included in all models as covariates. Folate intake was considered as a potentially important confounder given the key role of folate in the methyl metabolism pathway and demonstrated DNA hypomethylation in response to folate deficiency [57].

The following categories of descriptive variables were used: current rotating night work (yes, no), duration of night work in years (≤ 10, >10-≤20, >20), smoking (never, current, past); number of cigarettes smoked per day among current smokers (1–4, 5–14, >15); smoking duration (tertiles: ≤22.6, >22.6-≤31, >31 years); packyears (tertiles: ≤10.4, >10.4 - ≤17, >17); average alcohol drinking, in drinks per week (≤0.25, >0.25-≤1, >1); lifetime duration of alcohol drinking, in years (≤20, >20-≤30, >30); drinkyears—cumulative measure of alcohol drinking, calculated as the sum of the average number of drinks multiplied by the duration of drinking for each age category from the age of 15 years up to the age at the interview, expressed as drinks per week*years (≤5, >5-≤10, >10); current alcohol abstinence (yes vs no); BMI (<25, ≥25-<30, ≥30kg/m^2^); WHR (≤0.85, >0.85); total physical activity in MET*hr/wk (≤155, >155- ≤220, >220); and recreational physical activity (none vs any).

The odds ratios with 95% confidence intervals were calculated for each level of the lifestyle characteristics in the total study population. The basic model included adjustment for age and folate intake, while the extended one included mutual adjustment for all the other lifestyle characteristics, to gain information about the importance of the covariates. Trends were tested for continuous descriptive variables.

We also examined ORs for the subgroups of women characterized by the system of work and the category of lifestyle factors in order to determine the combined effect of these two factors. Unfortunately the analyses testing interactions between lifestyle factors and current work system were generally underpowered and thus their results are not reported.

Considering the missing data for folate intake for 91 women (13% of total) we imputed the values based on estimates derived from the FFQ at baseline study (FFQ1) and at follow-up (FFQ2). To this end, the linear regression model was fitted with the total amount of folate intake (in grams) as the dependent variable, and the frequency of food item intake, according to the FFQ1, and the average folate intake per a given food item (in grams per 100 g of a given food item), determined via FFQ2, as the independent variables.

Statistical analyses were performed using R version 3.1.1 (Vienna, Austria) and STATA 11 (StataCorp LP).

## Results

The general characteristics of the study population by *BRCA1* and *BRCA2* methylation status are displayed in Table 1. The average age of the study participants was 49 years, and age showed inverse and significant association with BRCA2 methylation (β-coef. = -0.039, 95%CI:-.0.075–-0.003). The study included roughly equal proportions of nurses currently working according to rotating night system or on day shifts (49% vs 51%). There was a high rate (49%) of subjects with a long lifetime duration of night work (>20 years) in the total population (80.4% among night shift workers and 18.5% among current day workers). As much as 30.6% of the subjects reported current smoking, while 42.5% had never smoked, and 27% were ex-smokers. Of the current smokers, roughly 41% smoked more than 15 cigarettes per day, and the majority (78%) were long-term smokers (>20 years). The subjects generally reported drinking small amounts of alcohol, with an average of 0.6 drinks per week, and 5% reported current abstinence. As much as 63% of the subjects were overweight or obese (BMI>25), and abdominal obesity (WHR.0.85) was observed among 25% of the subjects. About 28% reported no leisure-time physical activity, while their total physical activity was high (221 MET*hr per week). In most of the women studied, the promoter regions of the *BRCA*1 and *BRCA*2 genes were unmethylated (78% and 82%, respectively). The subjects’ characteristics according to the current work system are provided in S1 Table. Current night workers were on average two years younger than current day workers (p<0.001). As many as 80% of current night workers reported more than 20 years of night work (vs. 19% among current day workers), while 49% of day workers reported night work of up to 10 years (vs 3% of current night workers). More current night workers than day workers reported current smoking (34.6% vs. 26.7% respectively), with higher frequency of smokers, who smoked for 20 years or longer among ever smokers (82.6% vs 73.0%), and higher number of the packyears (9.9 vs 8.5, p = 0.007). The total physical activity was higher among night workers (241 vs. 202 MET*hr per week, p<0.001), and recreational physical inactivity was more common among night workers(p = 0.02).

**Table 1:** Selected characteristics of the studied population of nurses and midwives in the cross-sectional study.

Characteristic	n = 710	*BRCA1*		*BRCA2*^b^	
		Methylated n(%) n = 153 (21.5)	Unmethylated n(%) n = 557(78.5)	Methylated n(%) n = 130(18.3)	Unmethylated n(%) n = 579(81.5)
Age (years), AM (SD)	49.3 (5.3)	48.7 (5.3)	49.4 (5.3)	48.3 (5.3)	49.4 (5.3)
Age β–coefficient, 95%CI^c^		0.169(-0.058–0.010)		-0.039(-0.075–0.002)	
System of work, n (%)					
Current rotating nights	347 (48.9)	78 (51.0)	269 (48.3)	66 (50.8)	281 (48.5)
Current day work	363 (51.1)	75 (49.0)	288 (51.7)	64 (49.2)	298 (51.5)
Duration of the night work in years, n (%)					
≤10	189 (26.6)	38 (24.8)	151 (27.1)	35 (26.9)	153 (26.4)
>10-≤20	175 (24.7)	41 (26.8)	134 (24.1)	31 (23.9)	144 (24.9)
>20	346 (48.7)	74 (48.4)	272 (48.8)	64 (49.2)	282 (48.7)
Smoking, n (%)					
never	302 (42.5)	60 (39.2)	242 (43.5)	54 (41.5)	248 (42.8)
past	191 (26.9)	35 (22.9)	156 (28.0)	35 (26.9)	155 (26.8)
current	217 (30.6)	58 (37.9)	159 (28.5)	41 (31.5)	176 (30.4)
Amount of smoked cigarettes per day among current smokers, n (%)					
1–4	30 (13.8)	8 (5.2)	22 (4.0)	6 (4.6)	24 (4.2)
5–14	98 (45.2)	28 (18.3)	70 (12.6)	21 (16.2)	77 (13.3)
≥15	89 (41.0)	22 (14.4)	67 (12.0)	14 (10.8)	75 (13.0)
Smoking duration among ever smokers in years^b^, n (%)					
≤22.6	119 (29.2)	27 (29.0)	92 (29.2)	26 (34.2)	93 (28.1)
>22.6-≤31.0	126 (31.0)	24 (25.8)	102 (32.4)	19 (25.0)	107 (32.3)
>31.0	162 (39.8)	42 (45.2)	120 (38.1)	31 (40.8)	130 (39.3)
Packyears, AM (SD)	9.2 (10.9)	10.3 (11.7)	8.9 (10.7)	9.2 (11.3)	9.1 (10.8)
Alcohol drinking (no of drinks/week), AM (SD)	0.6 (0.7)	0.6 (0.6)	0.6 (0.7)	0.6 (0.9)	0.6 (0.7)
Lifelong duration of alcohol drinking (years), AM (SD)	29.9 (8.1)	29.3 (8.2)	30.1 (8.0)	29.3 (7.8)	30.0 (8.1)
Drinkyears, AM (SD)	11.3 (14.9)	10.6 (9.9)	11.5 (16.0)	12.8 (19.6)	10.9 (13.7)
Current alcohol abstinence^b^, n (%)					
yes	35 (4.9)	10 (6.5)	25 (4.5)	5 (3.9)	30 (5.2)
no	671 (94.5)	142 (92.8)	529 (95.0)	124 (95.4)	546 (94.3)
BMI (kg/m^2^), n (%)					
<25	262 (36.9)	58 (37.9)	204 (36.6)	54 (41.5)	208 (35.9)
≥25 - <30	281 (39.6)	58 (37.9)	223 (40.0)	50 (38.5)	230 (39.7)
≥30	167 (23.5)	37 (24.2)	130 (23.3)	26 (20.0)	141 (24.4)
WHR^b^, n (%)					
≤0.85	529 (74.5)	109 (71.2)	420 (75.4)	101 (77.7)	427 (73.8)
>0.85	180 (25.4)	44 (28.8)	136 (24.4)	29 (22.3)	151 (26.1)
Total physical activity (MET*hrs/wk), AM (SD)	221.2 (85.7)	215.8 (80.4)	222.7 (87.2)	221.3 (78.7)	221.2 (87.3)
Recreational PA^b^, n (%)					
None	198 (27.9)	36 (23.5)	162 (29.1)	38 (29.2)	160 (27.6)
Any	510 (71.8)	117 (76.5)	393 (70.6)	92 (70.8)	417 (72.0)
Total folate intake per day in μg AM (SD)	382 (138.5)	384.6 (139.0)	381.3 (138.5)	384.1 (137.5)	381.3 (138.9)
Total folate intake–β coefficient per 100 μg, 95%CI^c^		0.027(-0.099–0.154)		0.085(-0.0458–0.216)	

^a^—abbreviations: BMI—Body Mass Index; WHR–Waist to Hip Ratio; MET—Metabolic Equivalent

b—missing data for alcohol abstinence for 4 women; WHR, and WHtR for 1 woman, for recreational activity for 2 women, for methylation index in BRCA2 for 1 woman, smoking duration for 1 women

^c^–derived from univariate logistic regression

Neither the current night work status nor night work duration showed any association with the methylation status of the promoter site in the *BRCA*1 gene (Table 2). Of the lifestyle factors, smoking was found to be significantly associated with *BRCA*1 methylation, with OR = 1.52, 95%CI: 1.00–2.30 among current smokers compared to never-smokers. This results was slightly attenuated in the model with the other lifestyle factors included as covariates. No clear pattern was found for the relationship between the number of cigarettes smoked per day among current smokers and the methylation status of *BRCA*1. The OR was increased significantly among the subjects smoking 5–14 cigarettes per day (OR = 1.69, 95%CI:1.03–2.80), but not among those who reported smoking more than 15 cigarettes per day (p- trend = 0.053). The OR of methylated BRCA1 was significantly increased among those who reported smoking longer than 31 years (OR = 1.83, 95CI: 1.08–3.13), with a trend of borderline significance (p = 0.070). The relationship between the the number of packyears and the methylation of the promoter of *BRCA*1 was slightly weaker, with the OR = 1.55, 95%CI:0.97–2.47 for the highest tertile and p for trend of 0.082. BMI and other anthropometric measures were not associated with the methylation status of the *BRCA*1 gene. Neither night work nor smoking or alcohol drinking showed any association with the methylation status of the *BRCA*2 gene (Table 3). Likewise, no effect for the methylation status of *BRCA*2 could be noted for obesity or physical inactivity in the total study population.

**Table 2:** The association between methylation of *BRCA1* gene and lifestyle factors among nurses and midwives in the cross-sectional study.

	Basic model OR^a^, 95%CI	Model 1 OR, 95%CI
System of work		
Day work (reference)	1.00	1.0
Rotating nights	1.07 (0.74–1.54)	1.08 (0.73–1.59) ^b^
Duration of the night work in years		
≤10 (reference)	1.00	1.00
>10-≤20	1.19 (0.72–1.96)	1.18 (0.71–1.96) ^b^
>20	1.07 (0.65–1.66)	1.04 (0.66–1.64) ^b^
*p-trend*	*0*.*918*	*0*.*908*
Smoking status		
Never (reference)	1.00	1.00
Former	0.94 (0.59–1.50)	0.93 (0.58–1.50) ^c^
Current	1.52 (1.00–2.30)	1.50 (0.98–2.29) ^c^
Amount of smoked cigarettes per day currently		
Current non smoker(reference)	1.00	1.00
1–4	1.57 (0.67–3.63)	1.62 (0.69–3.80) ^c^
5–14	1.72 (1.05–2.83)	1.69 (1.03–2.80) ^c^
≥15	1.38 (0.81–2.35)	1.36 (0.79–2.34) ^c^
*p-trend*	*0*.*047*	*0*.*053*
Smoking duration (in years)		
0 (reference)	1.00	1.00
≤22.6	0.99 (0.59–1.66)	0.95 (0.56–1.61) ^c^
>22.6-≤31.0	1.13 (0.70–1.83)	1.15 (0.71–1.87) ^c^
>31.0	1.83 (1.09–3.08)	1.83 (1.08–3.13) ^c^
*p-trend*	*0*.*073*	*0*.*070*
Packyears		
0 (reference)	1.00	1.00
>0-≤10.4	1.18 (0.71–1.98)	1.15 (0.58–1.94) ^c^
>10.4-≤17.0	0.99 (0.58–1.68)	0.96 (0.56–1.64) ^c^
>17.0	1.50 (0.95–2.38)	1.55 (0.97–2.47) ^c^
*p-trend*	*0*.*090*	*0*.*082*
Alcohol drinking(no of drinks/wk)		
0-(reference)	1.00	1.00
>0–0.25	0.58 (0.26–1.29)	0.56 (0.25–1.24) ^d^
>0.25 –≤ 1	0.80 (0.36–1.77)	0.74 (0.33–1.65) ^d^
>1	0.63 (0.26–1.53)	0.59 (0.24–1.43) ^d^
*p-trend*	*0*.*763*	*0*.*741*
Lifelong duration of alcohol drinking (in years)		
-20 (reference)	1.00	1.00
>20-≤30	0.75 (0.38–1.49)	0.72 (0.36–1.43) ^d^
>30	0.74 (0.38–1.45)	0.67 (0.33–1.32) ^d^
*p-trend*	*0*.*715*	*0*.*482*
Drinksyears(drinks/wk*years)		
0–5 (reference)	1.00	1.00
>5-≤10	0.99 (0.63–1.57)	0.90 (0.56–1.44) ^d^
>10	1.02 (0.67–1.54)	0.91 (0.59–1.41) ^d^
*p-trend*	*0*.*579*	*0*.*427*
Current alcohol abstinence		
yes (reference)	1.00	1.00
no	0.67 (0.31–1.43)	0.62 (0.29–1.35) ^d^
BMI(kg/m^2^)		
<25 (reference)	1.00	1.00
≤25 - <30	0.95(0.63–1.45)	0.89 (0.59–1.37) ^e^
≥30	1.08(0.67–1.74)	1.06 (0.66–1.72) ^e^
*p-trend*	*0*.*712*	*0*.*668*
WHR		
≤0.85 (reference)	1.00	1.00
>0.85	1.39 (0.92–2.09)	1.32 (0.87–2.01) ^e^
*p-trend*	*0*.*930*	*0*.*620*
WHtR		
≤0.6 (reference)	1.00	1.00
>0.6	0.98(0.61–1.52)	0.96 (0.61–1.51) ^e^
*p-trend*	*0*.*800*	*0*.*875*
Total physical activity (MET*hrs/wk)		
≤155 (reference)	1.00	1.00
>155- ≤220	1.00(0.62–1.64)	1.00 (0.60–1.65) ^f^
>220	0.84(0.52–1.36)	0.81 (0.49–1.34) ^f^
*p-trend*	0.057	*0*.*051*
Recreational PA		
None (reference)	1.00	1.00
Any	1.34(0.88–2.04)	1.34 (0.87–2.05) ^f^

^a^—adjusted for age and folate intake

b—adjusted for age, folate intake, current smoking, drinks per week, BMI and total PA

c–adjusted age, folate intake, drinks per week, BMI and total PA

^d^–adjusted for age, folate intake, current smoking, BMI and total PA

^e^–adjusted for age, folate intake, current smoking, drinks per week and total PA

^f^–adjusted for age, folate intake, current smoking, drinks per week and BMI

**Table 3:** The association between methylation of *BRCA2* gene and lifestyle factors among nurses and midwives in the cross-sectional study.

	Basic model OR^a^, 95%CI	Model 1 OR, 95%CI
System of work		
Day work (reference)	1.00	1.00
Rotating nights	1.02 (0.69–1.51)	1.09 (0.72–1.65)^b^
Duration of the night work in years		
≤10 (reference)	1.00	1.00
>10-≤20	0.91 (0.53–1.56)	0.94 (0.55–1.63)^b^
>20	0.97 (0.61–1.54)	1.02 (0.64–1.64)^b^
*p-trend*	*0*.*985*	*0*.*778*
Smoking status		
Never (reference)	1.00	1.00
Former	1.10 (0.68–1.77)	1.09 (0.97–1.77)^c^
Current	1.12 (0.71–1.76)	1.11 (0.70–1.77) ^c^
Amount of smoked cigarettes per day currently		
Current non smoker(reference)	1.00	1.00
1–4	1.19 (0.47–3.02)	1.20 (0.47–3.08) ^c^
5–14	1.30 (0.76–2.22)	1.27 (0.74–2.84) ^c^
≥15	0.84 (0.45–1.56)	1.05 (0.69–1.62) ^c^
*p-trend*	*0*.*970*	*0*.*958*
Smoking duration (in years)		
0 (reference)	1.00	1.00
≤22.6	1.12 (0.66–1.89)	1.09 (0.64–1.86) ^c^
>22.6-≤31.0	0.88 (0.52–1.50)	0.89 (0.52–1.51) ^c^
>31.0	1.55 (0.89–2.73)	1.55 (0.88–2.76) ^c^
*p-trend*	*0*.*338*	*0*.*340*
Packyears		
0 (reference)	1.00	1.00
>0-≤10.4	1.30 (0.77–2.20)	1.25 (0.73–2.14) ^c^
>10.4-≤17.0	0.87 (0.49–1.54)	0.85 (0.48–1.52) ^c^
>17.0	1.20 (0.73–1.97)	1.23 (0.73–2.02) ^c^
*p-trend*	*0*.*607*	*0*.*544*
Alcohol drinking(no of drinks/wk)		
0-(reference)	1.00	1.00
>0–0.25	1.41 (0.52–3.81)	1.38 (0.51–3.77) ^d^
>0.25 –≤ 1	1.42 (0.52–3.90)	1.37 (0.50–3.80) ^d^
>1	1.52 (0.52–4.47)	1.49 (0.50–4.42) ^d^
*p-trend*	*0*.*287*	*0*.*279*
Lifelong duration of alcohol drinking (in years)		
-20 (reference)	1.00	1.00
>20-≤30	0.81 (0.39–1.70)	0.85 (0.51–1.43) ^d^
>30	0.93 (0.45–1.95)	1.23 (0.78–1.94) ^d^
*p-trend*	*0*.*807*	*0*.*926*
Drinksyears(drinks/wk*years)		
0–5 (reference)	1.00	1.00
>5-≤10	0.88 (0.53–1.46)	0.78 (0.37–1.64) ^d^
>10	1.27 (0.82–1.97)	0.89 (0.42–1.89) ^d^
*p-trend*	*0*.*153*	*0*.*173*
Current alcohol abstinence		
yes(reference)	1.00	1.00
no	1.43 (0.54–3.80)	1.39 (0.52–3.73) ^d^
BMI(kg/m^2^)		
<25 (reference)	1.00	1.00
≤25 - <30	0.89 (0.57–1.37)	0.86 (0.55–1.34)^e^
≥30	0.79 (0.46–1.32)	0.77 (0.46–1.30) ^e^
*p-trend*	*0*.*089*	*0*.*096*
WHR		
≤0.85 (reference)	1.00	1.00
>0.85	0.87 (0.53–1.37)	0.89 (0.56–1.42) ^e^
*p-trend*	*0*.*993*	*0*.*942*
WHtR		
≤0.6 (reference)	1.00	1.00
>0.6	0.68 (0.40–1.12)	0.70 (0.42–1.16) ^e^
*p-trend*	*0*.*510*	*0*.*512*
Total physical activity (MET*hrs/wk)		
≤155 (reference)	1.00	1.00
>155- ≤220	0.92 (0.55–1.56)	0.89 (0.52–1.53)^f^
>220	0.87 (0.53–1.45)	0.84 (0.50–1.43)^f^
*p-trend*	*0*.*451*	*0*.*542*
Recreational PA		
None (reference)	1.00	1.00
Any	0.93 (0.61–1.43)	0.89 (0.58–1.36)^f^

^a^—adjusted for age and folate intake

b—adjusted for age, folate intake, current smoking, drinks per week, BMI and total PA

c–adjusted age, folate intake, drinks per week, BMI and total PA

^d^–adjusted for age, folate intake, current smoking, BMI and total PA

^e^–adjusted for age, folate intake, current smoking, drinks per week and total PA

^f^–adjusted for age, folate intake, current smoking, drinks per week and BMI

## Discussion

In the reported cross-sectional study of nurses and midwives, we examined several determinants of the methylation status in two suppressor genes, namely *BRCA*1 and *BRCA*2, whose silencing predisposes to breast cancer. We sought associations for the rotating night work, cigarette smoking, alcohol consumption, physical activity and two anthropometric measures: BMI and WHR. The study found no associations for rotating night shift while suggested a positive association between the methylation status of the *BRCA*1 gene and current smoking, particularly among moderate smokers (5–14 cigarettes per day), and long term smokers (>31 years). No other significant associations were noted.

Although the research concerning the epigenetic effects of smoking, mostly in the context of cancer (e.g. lung cancer for which smoking is a strong risk factor), has been relatively abundant, we were able to identify only a few studies focused on associations between smoking and the methylation status [58] within the promoter regions of *BRCA*1, and in fact none for *BRCA*2. In a recent study conducted among monozygotic twins (21 couples) with discordant smoking habits, no significant difference in the methylation status of the promoter site of *BRCA*1 was noted between smokers and nonsmokers [59]. The odds ratio of *BRCA*1 promoter hypermethylation was increased among the subjects who smoked for more than 10 years (OR = 1.71, 95%CI: 0.09–31.9) but the result was insignificant. The study was limited by its relatively small population size, including mostly light smokers (<15 cigarettes per day) with a short duration of the smoking habit (mean 10 years). In another study, neither active smoking (current or ever) nor passive smoking was related to the methylation status within the promoter site of *BRCA*1 when measured in tumor tissue from breast cancer cases [60]. A number of epigenome-wide studies reported differential methylation of various loci across genome between smokers and non-smokers, although none of them identified statistically significant variability in the methylation pattern by the smoking status for the *BRCA*1 or *BRCA*2 genes [61–66]. The analyses of data from the genome-wide studies imply strong thresholds for significance; thus smaller effects may be excluded from being quantified. For instance, these studies did not find associations for smoking and methylation in the promoter region of another suppressor gene–*CDKN2A (p16)*, while a meta-analysis of 19 epidemiological studies using the candidate genes approach demonstrated a higher frequency of the *p16* gene hypermethylation in smoking vs non-smoking patients in tumor tissues of patients with diagnosed non-small cell lung carcinoma (OR = 2.25, 95%CI:1.81–2.80) [67].

The biological mechanism explaining the relations between smoking and epigenetic effects in particular gene-specific hypo- or hypermethylation remains unclear. Several mechanisms were postulated, mostly related to the activity of DNA methyltransferases, the enzymes responsible for DNA methylation. The carcinogens contained in cigarette smoke, such as polycyclic aromatic hydrocarbons, chromium and formaldehyde, are well known to generate DNA damage including double-strand breaks [68–70]. It was also shown that the DNA repair sites recruit DNA methyltransferase 1 (DNMT1), resulting in the methylation of the repaired DNA fragments [71–74]. Also cadmium, contained in cigarette smoke, was demonstrated to alter DNA methylation pattern through cadmium-mediated overexpression of *DNMT3B* with concomitant hypermethylation of the tumor suppressor genes *p16* and *RASSF1A* [75]. Changes in DNA methylation were related to histone modification caused by cigarette smoking [76], which correlated with decreased *DNMT1* and increased *DNMT3B* expression along with hypomethylation of repetitive DNA sequences and hypermethylation of tumor suppressor genes *RASSF1A* and *RARB* [76].

The epidemiological data regarding other environmental determinants of the epigenetic events in *BRCA*1 and *BRCA*2 have been sparse and inconclusive thus far. To our knowledge, no previous study that focused on these two genes examined the possible associations with night work. Two broad studies on epigenome investigated associations for the night work [44,47], but only the latter reported findings for the *BRCA*1 gene [47]. In this study, as much as 65% of the investigated sites showed hypomethylation in night workers compared to day workers. The hypomethylation referred to the sites (cytosines) within the *BRCA*1 gene (both within the promoter region and the gene body) [47]. This was the only study that showed that night shift work is associated with hypomethylation in the investigated genomic DNA, while in other studies in this field, both hypo- and hypermethylation was observed, depending on the gene or the gene region studied [44,45,77].

We found no significant associations between BMI or WHR and the *BRCA*1 or *BRCA*2 methylation status in the total study population. In one previous report, BMI was significantly associated with methylation in the *BRCA*1 gene determined in DNA extracted from breast tumor tissue [78]. This observation has not been supported by two other studies that investigated the methylation pattern of *BRCA*1 besides other cancer-related genes in breast tumor tissue [79]. The inconsistency of the findings may be explained by a possible mismatch of loci within the *BRCA*1 gene that were examined in each of these studies, as suggested by McCullough et al. [79]. An epigenome-wide association study of the methylation pattern of DNA deriving from peripheral white blood cells did not report inferences between BMI and the methylation pattern in the *BRCA*1 or *BRCA*2 gene [80].

We also did not find associations between the methylation status of both the examined genes and the physical activity in the total study population. Previous research demonstrated that physical activity may be associated with a “healthier methylation profile”[81,82]. As far as we know, there was only one study addressing this specific association for the genes we investigated. The intervention trial examined methylation level across 45 sites within a panel of 21 breast cancer-related genes (including 4 sites in *BRCA*1 and 1 site in *BRCA*2) [81]. This study demonstrated lower levels of methylation among individuals who exercised more minutes per week and also among those who increased their physical activity during 12 months of the trial. The most recent review evaluated data from 25 studies (both observational and intervention based) addressing association between physical activity and methylation pattern [83]. The authors concluded that both acute and chronic exercising significantly influence DNA methylation. The biological mechanisms of the process remain to be elucidated [83].

In our study we analyzed the main effect of the factors on *BRCA1* and *BRCA2* genes methylation with two models. The comparison of results from these two models showed no substantial differences, hence no strong confounding was detected. The present project was a pioneer study in the field of epigenetics within two important breast cancer-related genes as analyzed by night shift work, thus contributing to the sparse epidemiological data about inferences for modifiable lifestyle factors, and this can be regarded as one of its strong points. The size of the study population was relatively large when compared to other cross-sectional studies searching for mechanistic explanation of increased breast cancer risk among night shift workers. We also obtained detailed exposure data through in-person interviews and anthropometric measurements. The analyses were focused and respective hypotheses were formulated. The qMSP used for determining the *BRCA1* and *BRCA2* methylation patterns bears a high degree of sensitivity. This highly quantitative technique can accurately determine the relative prevalence of a particular pattern of CpG dinucleotides methylation.

There are also several limitations to consider. The conclusions regarding occupational exposure are confined to the rotating night shift work, therefore the results cannot be generalized to other shift work systems. The part of analysis referring to alcohol consumption was limited by the fact that in the population we studied, the level of alcohol consumption was generally low. Only 6 women reported having more than 5 drinks per week. Thus we were not able to examine associations for heavy drinkers. Even though the total study population was relatively large, the number of subjects in specific categories in some instances was rather small (below 10). This resulted in wide confidence intervals, which was the case e.g. for the shortest duration of night work history among current night workers (<10 years), light current smokers (1–4 cigarettes per day), or ever-smokers with a shorter smoking duration (<10 years).

In the analysis, we included the age and folate intake and other lifestyle characteristics that were considered in this study. While we could not rule out some confounding by other factors, we are not aware of any strong determinants of de novo methylation in *BRCA*1 or *BRCA*2.

Apart from the limitations mentioned above, the utility of peripheral blood for the assessment of associations between the risk factors and methylation alterations in genomic DNA may have had impact, given that the sample does not directly reflect the target tissue, as it has been indicated for human blood and brain using 450K Human Methylation array[84]. On the other hand, 4 of 8 investigated CpG loci showed a good correlation between blood and buccal cells in DNA methylation patterns [85].

There are only a few studies that focus on the potential reflection of the epigenetic status in DNA from the surrogate tissues compared with DNA from breast tumor or normal tissue. Fu et al and Sharma et al noted a concordance between tumor DNA methylation in breast cancer patients and paired serum or plasma DNA methylation of multiple cancer genes [86,87]. These findings indicate that particular DNA methylation patterns in peripheral blood cells may be useful for predicting breast cancer risk. However, no correlation was found between white blood cell DNA and normal breast epithelial cell DNA methylation of *RASSF1* tumor suppressor gene and repetitive elements in healthy women undergoing reduction mammoplasty [88]. It has also been postulated that breast cancer treatment may potentially affect DNA methylation which would imply that the epigenetic analyses should ideally be conducted in untreated patients [50,51].

Nevertheless, blood-derived DNA methylation seems to be a promising epigenetic indicator in breast cancer investigations. In a recent systematic review, a possibility of using blood-derived DNA methylation features (global methylation or gene-specific methylation) for breast cancer stratification was suggested. The most frequently investigated gene in whole blood was *BRCA1*, which presented a higher methylation status in the promoter region in patients compared to controls [89]. Of concern is also that DNA methylation may vary depending on the white blood cell type [84]. Tissue-specific methylation patterns are established through a combination of demethylation and de novo methylation reactions [90]. Tissue specificity may be due to the differences in specific DNA binding proteins that bind to methylated and unmethylated DNA, and the availability of DNA methylase, all of which may play a role in the extent of de novo methylation or demethylation. Using the total population of mono- and polymorphonuclear white blood cells for DNA isolation may affect the methylation pattern of the genes. However, it has been shown that the majority of CpG loci tested presented stable methylation over a long time (11–20 years) in DNA isolated from blood taken at two time points, despite the possible changes in the cellular morphology of blood [85]. Thus considering the absence of temporal variation in the methylation pattern, we are of the opinion that specific gene-methylation can be reliably assessed using one blood sample.

Another limitation refers to the methodology for determining the methylation status. We did not examine all the epigenetic events in the CpG island within the whole promoter region, but only the 11CpG dinucleotides in *BRCA1* and 10 in *BRCA2*.

The question also arises whether the storage time may affect the methylation pattern. It has recently been shown that most of the storage conditions for blood specimens have no effect on DNA integrity and methylation [91]. Thus it seems reasonable that the covalent binding of a methyl group to cytosines in gDNA samples obtained from venous blood and preserved for 6–7 years, since 2008 were not affected by the storage period. One of the mechanisms that may have influence on the stability of the methylation patterns is the formation of oxidative DNA bases such as 8-oxo-guanine, which is present in the CpG dinucleotides [91]. However, the rate of formation of this modified base in sub-freezing temperatures used for the storage of blood samples is negligible.

In conclusion, our study did not detect associations between the methylation index within the promoter sites of *BRCA1* and *BRCA2* genes and the rotating night shift work. The results of our research indicate that smoking may be one of the environmental determinants of the methylation status of the promoter region in the *BRCA*1 gene. Given the novelty of these findings, and limitations resulting from the relatively small number of subjects in subgroup analyses further validation research is warranted.

## Supporting information

S1 Table Selected characteristics of the studied population of nurses and midwives in the cross-sectional study by current rotating night work status. (PDF)
