# Supplementary material for: Rotating night work, lifestyle factors, obesity and promoter methylation in BRCA1 and BRCA2 genes among nurses and midwives
Source: PLoS One. 2017 Jun 8;12(6):e0178792. doi: 10.1371/journal.pone.0178792 (PMC5464581; doi:10.1371/journal.pone.0178792)
Supplement: S1 Table — (PDF) [file pone.0178792.s001.pdf]

S1 Table. Selected characteristics of the studied population of nurses and midwives in the cross-sectional study by current rotating night work status

Characteristic	Current rotating night workers N=347	Current day workers N=363	P[d]
Age (years), AM (SD)	48.3(5.2)	50.2(5.3)	<0.001[c]
Duration of the night work in years , n (%)			
≤10	10(2.9)	179(49.3)	<0.001
>10-≤20	58(16.7)	117(32.2)	
>20	279(80.4)	67(18.5)	
Smoking, n (%)			
never	146(42.1)	156(43.0)	0.07
past	81(23.3)	110(30.3)	
current	120(34.6)	97(26.7)	
Amount of cigarettes smoked per day among current smokers, n (%)			
1-4	0	0	0.27
5-14	16(13.3)	14(14.4)	
15-24	49(40.8)	49(50.5)	
>24	55(45.8)	34(35.1)	
Smoking duration among ever smokers in years, n[b] (%)			
≤10	16(7.9)	32(15.5)	0.004
>10-≤20	19(9.5)	23(11.1)	
>20	166(82.6)	151(73.0)	
Packyears, AM (SD)	9.9(11.3)	8.5(10.5)	0.007
Alcohol drinking(no of drinks/week), AM (SD)	06(0.7)	0.6(0.7)	0.56
Lifelong duration of alcohol drinking (years), AM (SD)	29.2(8.0)	30.5(8.1)	0.94
Drinkyears, AM(SD)	11.2(15.1)	11.3(14.8)	0.83
Current alcohol abstinence, n(%)			
Yes	19(5.5)	16(4.4)	0.50
No	328(94.5)	347(95.6)	
BMI(kg/m^2) , n(%)			
<25	124(35.7)	138(38.0)	0.70
≥25 - <30	137(39.5)	144(39.7)	
≥30	86(24.8)	81(22.3)	
WHR[b], n (%)			
≤0.85	255(73.5)	274(75.5)	0.11
>0.85	91(26.2)	89(24.5)	
Total physical activity (MET*hrs/wk), AM (SD)	241(78)	202(88)	<0.001
Recreational PA[b], n(%)			
None	111(32.0)	87(24.0)	0.02
Any	235(67.7)	275(75.8)	
Total folate intake per day in µg AM (SD)	381.3(136.6)	382.7(140.5)	0.37
Methylation index of the promoter of *BRCA1* gene, n (%)			
unmethylated	269(77.5)	288(79.3)	0.73
methylated	78(22.5)	75(20.7)	
Methylation index of the promoter of *BRCA2* gene[b], n (%)			

unmethylated	281(81.0)	298(82.1)	0.93
methylated	66(19.0)	64(17.6)	

[a] - abbreviations: BMI - Body Mass Index; WHR – Waist to Hip Ratio; MET - Metabolic Equivalent

[b] – missing data for WHR ,and WHtR for one day worker; for smoking duration in 1 woman day worker; for recreational activity for 1 day and 1 rotating night worker, for global methylation for 4 day and 3 rotating night workers, , for methylation index in BRCA2 for 1 day worker

[c] – p determined with t test

[d] – p determined with regression analysis age adjusted
